# Arts and Health Glossary - A Summary of Definitions for Use in Research, Policy and Practice

**DOI:** 10.3389/fpsyg.2022.949685

**Published:** 2022-07-22

**Authors:** Christina R. Davies, Stephen Clift

**Affiliations:** ^1^School of Allied Health, University of Western Australia, Perth, WA, Australia; ^2^Sidney De Haan Research Centre for Arts and Health, Canterbury Christ Church University, Canterbury, United Kingdom

**Keywords:** art, arts, health, glossary, evidence, definition, research, policy

## Introduction

Evidence-based research, reviews, policy, practice and programs, informed by the discipline of ‘*Arts and Health*' (also sometimes referred to as ‘Arts in Health,' ‘Arts for Health,' ‘Arts-Health') (White, 2009), have the potential to positively contribute to the *health* and *wellbeing* of the general population and specific population groups (e.g., young people, older adults, LGBTQI+ people, refugees, people with a disability, people who are isolated, etc) (Smith, 2002; South, 2004; Staricoff, 2004; Putland, 2008; White, 2009; Fraser et al., 2015; Mapuana et al., 2015; Menzer, 2015; Clift and Camic, 2016; Davies et al., 2016; Wreford, 2016; Zarobe and Bungay, 2017; Daykin et al., 2018; A New Approach (ANA), 2019; Vella-Burrows et al., 2019; Davies and Pescud, 2020; Corbin et al., 2021). A recent systematic review of both qualitative and quantitative articles found ‘strong evidence' of the impact of arts engagement on mental wellbeing, ‘moderate to strong evidence' on social health and ‘emerging/low evidence' related to healthy eating, physical activity, preventing tobacco use and preventing harm from alcohol (Davies and Pescud, 2020). Although the idea that the arts can impact health is not novel (e.g., paintings have been used in hospitals since the middle ages to enhance the health environment) (Clift et al., 2009), compared to other health fields, the discipline of Arts and Health is relatively new, therefore a glossary of definitions is useful to facilitate communication and to clarify terminology and concepts from which evidence-based research, reviews, policy, practice and programs can be developed. The definitions included in this opinion paper are not intended to be exhaustive and draw on a wide range of disciplines including health promotion, epidemiology, psychology, medicine and the arts. We have endeavored to keep our definitions short, and where needed, encourage the reader to seek deeper interpretations and explanations which may be found by consulting the relevant references associated with each definition. When reading this opinion piece, we encourage the reader to consider the following limitations. First, some of the concepts and definitions used in this glossary reflect the discipline of expertise, experience, cultural bias and country of the authors (i.e., Australia and the UK). Second, the definitions provided will be influenced by current language, knowledge, health, social and economic conditions. Third, the definitions provided are by their very nature summaries of complex ideas and therefore restrictive in scope. With these limitations in mind, an arts and health glossary on which to base shared language and meaning, still has the potential to facilitate understanding, co-operation and multi-discipline partnerships at a local, national and international level.

## Arts and Health Glossary

### Arts

**Figure 1:**
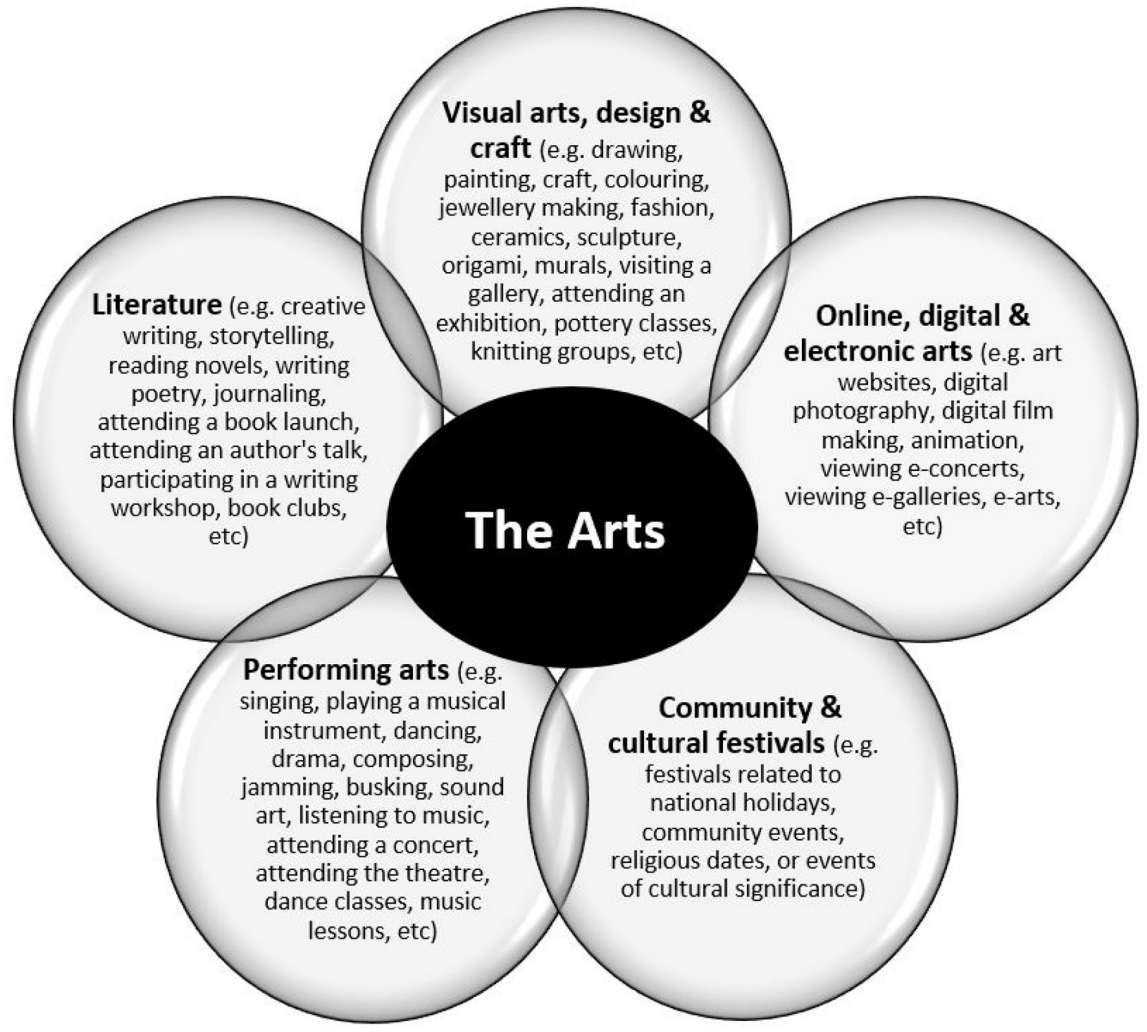
Art forms, activities and events (adapted with permission, Davies et al., 2012).

The *Arts* is an umbrella term. Using a consultative approach, a definition of the *Arts* was developed by contacting experts in the field of the arts or arts-health from the UK, Australia, Europe, USA and Canada (*n* = 280, 44% response) (Davies et al., 2012). A person was considered to be an expert if they were a director, manager or curator of a leading arts organization, or an academic who had published a major arts/arts–health report or journal article (Davies et al., 2012). Based on expert knowledge and informed opinion, the *Arts* were defined by five main art forms and (within these art forms) a comprehensive list of activities and events which articulated the numerous ways people engaged in the arts. These five art forms are listed below and detailed in Figure 1: (Davies et al., 2012)

- Performing arts (e.g., active and receptive activities in the genre of music, dance, singing, drama, sound art, etc);
- Visual arts, design and craft (e.g., active and receptive activities in the genre of painting, drawing, craft, jewelery, ceramics, sculpture, fashion, textiles, etc);
- Community and cultural festivals (e.g., active and receptive activities in the genre of festivals such as Diwali festivals, community lantern festivals, Lunar New Year festivals, etc);
- Literature (e.g., active and receptive activities in the genre of storytelling, creative writing, journaling, publishing, etc), and
- Online, digital and electronic arts (e.g., active and receptive activities in the genre of animation, digital photography/film, e-arts, arts websites, arts related social media, e-galleries, etc).

It should be noted that each art form may operate independently, but may also collaborate, communicate and intersect in their arts practice with the other four art forms.

### Arts Engagement

*Arts engagement* (also referred to as recreational arts engagement) is an umbrella term (Archibald and Kitson, 2019; Davies and Pescud, 2020) that describes the various ways individuals interact with the arts as part of their everyday life for enjoyment, entertainment, socially, as a hobby or as part of an organized program. Methods of engagement include (but are not limited to) making, creating, learning, performing, participating, attending, experiencing, listening to, and viewing art (Davies et al., 2012). Arts engagement may be undertaken individually (i.e., by yourself) or with others (e.g., friends, family, other participants). Arts engagement occurs within a variety of *settings* and on a continuum from *active engagement* to *receptive engagement* (Davies et al., 2016) for example:

- ***Active arts engagement***: includes overtly or directly making, performing or creating art (e.g., performing in a concert as a musician, singer or dancer; painting a picture, writing a poem, making a movie),
- ***Receptive arts engagement***: includes experiencing, attending, listening or viewing art (e.g., attending a concert as part of an audience or online audience, listening to music, viewing a painting in a gallery or e-gallery, reading a poem, watching a movie).

When a person plays a musical instrument, listens to music, sings, dances, paints, draws, reads a novel, writes creatively, attends a festival, gallery or takes part in an art class, program or intervention with an artist, musician, actor, dancer, singer, etc this is arts engagement. It is important to note that *arts engagement* and *art therapies* are not the same thing (e.g., music therapy, art therapy, drama therapy, dance movement therapy, etc) (American Art Therapy Association, 2017; The British Association of Dramatherapists, 2020; American Music Therapy Association, 2022; Dance Movement Therapy Association of Australasia, 2022). For example, dancing while at a festival with family and friends for fun, enjoyment and entertainment (arts engagement), is not the same as engaging in dance movement therapy in a treatment environment with a qualified therapist for remedial or diagnostic purposes.

### Arts and Health

*Arts and Health* includes arts engagement and art therapies (Meeting of Cultural Ministers (MCM), 2013), and broadly refers to: (Davies and Pescud, 2020)

- The practice of applying *arts* initiatives in a variety of *settings* to directly promote, maintain or improve health and wellbeing outcomes (e.g., arts programs to improve the mental, social and/or physical health of participants); and/or
- The introduction of works of art (e.g., paintings, music, sculptures, etc.) into a *setting* to enhance health and wellbeing in that environment (e.g., music in waiting rooms to enhance patient mood); and/or
- The practice of applying *health or wellbeing* initiatives in arts settings, venues or events to promote, maintain or improve health outcomes (e.g., water provided for free at festivals especially if alcohol is also available; healthy food options provided by vendors during an arts event).

### Arts on Prescription

*Arts on prescription* is a form of social prescribing (Poulos et al., 2019). Social prescribing enables health practitioners to refer patients to a range of local, non-clinical programs and services to address a range of psychosocial and socioeconomic issues (Torjesen, 2016; Chatterjee, 2018; The Royal Australian College of General Practitioners Consumers Health Forum of Australia, 2019; NHS England, 2022). Arts on prescription, involves a referral process to an experienced artist (rather than a therapist) who facilitates arts activities (e.g., painting, drama, craft, photography, dance, singing, lantern making, etc) in a group setting, to positively impact participant wellbeing (Bungay and Clift, 2010; Poulos et al., 2019). The purpose of arts on prescription is not to replace conventional medical treatment but rather to act as an adjunct or complement to promote wellbeing, creativity and social engagement (Bungay and Clift, 2010).

### Art Therapies

*Arts engagement* and *art therapies* are not the same thing. Art therapies (e.g., music therapy, art therapy, drama therapy, dance movement therapy, etc) (American Art Therapy Association, 2017; The British Association of Dramatherapists, 2020; American Music Therapy Association, 2022; Dance Movement Therapy Association of Australasia, 2022) are an “*integrated mental health and human services profession*” (American Art Therapy Association, 2017). Art therapies are a form of psychotherapy, that involve a therapeutic relationship between a qualified therapist and an individual who engage in creative activities for diagnostic or remedial purposes (American Art Therapy Association, 2017; The British Association of Dramatherapists, 2020; American Music Therapy Association, 2022; Dance Movement Therapy Association of Australasia, 2022) *Arts engagement* however, is something that people do as part of their everyday life (by themselves or with others) for enjoyment, entertainment, socially, as a hobby or as part of an organized program. When a person plays a musical instrument, listen to music, sings, dances, paints, draws, read a novel, writes creatively, attend a festival, gallery or takes part in an art class, program or intervention with an artist, musician, actor, dancer, singer, etc (rather than a therapist) this is arts engagement – not *art therapy/therapies* (Davies et al., 2016).

### Evidence-Based Arts and Health

Arts and health policy, practice and organized programs should be guided by evidence-based research and evaluation (qualitative and quantitative) that is rigorous, appropriate, systematic, trustworthy and transparent, rather than methods based on anecdote and opinion (Hamilton et al., 2003; Davies et al., 2012; Daykin and Joss, 2016; Clift et al., 2021). To avoid overstating cause-effect relationships or making erroneous statements of impact, when assessing or evaluating arts and health policies, practice and programs it is useful for practitioners and researchers to consider regional context, bias, precision, relevance, appropriateness and levels of evidence (e.g., in the research hierarchy, the level of evidence provided by a case study is different to that provided by a prospective cohort study or a randomized control trial) (NHMRC, 2008; Merlin et al., 2009; Hillier et al., 2011; Munn et al., 2014). In addition, when conducting a review, it is also important to consider appropriateness. For example a scoping review is appropriate when the purpose is to identify evidence types, clarify or map key concepts, identify knowledge gaps or act as a precursor to a systematic review; a systematic review however is appropriate when making policy, practice and funding decisions or when considering feasibility, quality, appropriateness, effectiveness or to refute/confirm current practices (Munn et al., 2018).

### Health

The World Health Organization (WHO) defines *health* as “*a state of complete physical, mental and social wellbeing and not merely the absence of disease or infirmity”* (World Health Organization, 1946). Since it was first articulated as part of the preamble to the constituent of the WHO, this definition has been regularly contested e.g., emphasis on the word “complete” suggests a level of wellbeing that would cause the majority of society to be categorized as unhealthy (Huber, 2011). Nevertheless, the positive, holistic and aspirational view of health as embracing different forms of wellbeing continues to inform much health, public health and health promotion practice.

### Health Behavior

A *health behavior* is an activity undertaken by a person, target group or population for the purpose of promoting, maintaining or improving their health and wellbeing (Nutbeam, 1998).

### Outcomes

In *health*, an outcome relates to a change in the health status (positive or negative) of a person, a target group or population related to a planned or unplanned activity, event, service, program, intervention, policy, regulation or law (Nutbeam, 1998). The ‘Healthy Arts Framework' (Davies et al., 2014) describes the relationship between arts engagement and both positive and negative health outcomes within the themes of mental health, social health, physical health, economic, knowledge, identity and art specific outcomes (Davies et al., 2014). The Healthy Arts Framework is guided by the biopsychosocial model of health (Engel, 1977), theories of social epidemiology (Krieger, 2001), positive psychology (Seligman and Csikszentmihalyi, 2000) and a salutogenic perspective of health (Mittelmark and Bauer, 2017). Within this framework, outcomes may be direct and deliberate (e.g., people with lung disease singing in a choir as part of their pulmonary rehabilitation), or health outcomes may be achieved unintentionally (e.g., a person attending a jewelery class to make necklaces, but the art class also increases their mental wellbeing and social interaction with others).

### Settings

A setting is a place or social context in which people engage in their everyday lives (Nutbeam, 1998). Arts engagement occurs within a variety of settings, including but not limited to the home, community centers, recreation centers, parks, schools, universities, workplaces, places of worship, aged care, hospitals, prisons, museums, theaters, concert halls, art galleries, online, etc (Davies et al., 2012).

### Wellbeing

Wellbeing is a multidimensional construct that broadly relates to how people experience, perceive or evaluate the quality or condition of their life (Maggino, 2015; Linton et al., 2016; Centers for Disease Control Prevention, 2018; Oman, 2021). Wellbeing is explained by a number of theories and is a complex combination of a person's physical health, social connection, cultural/spiritual connection, societal/civic engagement, economic influences (e.g., housing, work, wealth, work-life balance), knowledge/skills, sustainable development, the environment (e.g., quality, safety), emotional and mental health, and is not just about single factors such as happiness, or the absence of disease (Linton et al., 2016; Centers for Disease Control Prevention, 2018; Better Health, 2020; Jones et al., 2021; Oman, 2021; Office of the Future Generations Commissioner for Wales, 2022; Organisation for Economic Co-operation Development, 2022). Wellbeing can be both positive and negative and can be measured *via* subjective (e.g., asking people how they are feeling) or objective data (e.g., life expectancy, household income, economic growth) (Centers for Disease Control Prevention, 2018; Oman, 2021). Wellbeing is assessed in a number of ways, e.g., the OECD wellbeing indicators, (Organisation for Economic Co-operation Development, 2022) New Zealand's Wellbeing Budget, (New Zealand Government, 2022) the UK Measure of National Well-being, (Office for National Statistics, 2021) and *via* a large number of validated scales, (Linton et al., 2016) e.g., the Warwick-Edinburgh Mental Wellbeing Scale (WEMWBS), (Warwick Medical School, 2021) Ryff Psychological Wellbeing Scale, (Standford University–Department of Psychology, 2022) and the WHO-5 Wellbeing Index (Topp et al., 2015).

## Conclusion

Given the breadth of the arts and health discipline a glossary is a step toward shared understanding and meaning that can facilitate communication, policy, practice and research. As the field of arts and health grows and concepts evolve the definitions within this paper will need to be assessed and updated to ensure relevance. We hope this opinion piece will result in debate within the field and that despite the obvious restrictions of a glossary will provide a summary of key terminology, basic ideas and concepts central to the development of the arts and health discipline.

## Author Contributions

CD conceived the glossary and led the development. CD and SC contributed to the definitions, critical review, and final version of the glossary. All authors read and approved the final manuscript.

## Funding

This work was supported by The Ian Potter Foundation (Ref: 31110974) and The Minderoo Foundation – Arts & Culture (Ref: 2022/GR000916). The Ian Potter Foundation and The Minderoo Foundation are two of Australia's major philanthropic foundations. This work is also supported by the Western Australian Future Health Research and Innovation Fund, which is an initiative of the Western Australian State Government (Ref: TFMH2021-CD).

## Conflict of Interest

The authors declare that the research was conducted in the absence of any commercial or financial relationships that could be construed as a potential conflict of interest.

## Publisher's Note

All claims expressed in this article are solely those of the authors and do not necessarily represent those of their affiliated organizations, or those of the publisher, the editors and the reviewers. Any product that may be evaluated in this article, or claim that may be made by its manufacturer, is not guaranteed or endorsed by the publisher.
